# Comparison of Volatile Components between Raw and Vinegar Baked Radix Bupleuri by GC-MS Based Metabolic Fingerprinting Approach

**DOI:** 10.1155/2015/653791

**Published:** 2015-07-21

**Authors:** Jie Xing, Hui-Min Sun, Zhen-Yu Li, Xue-Mei Qin

**Affiliations:** ^1^Modern Research Center for Traditional Chinese Medicine of Shanxi University, No. 92, Wucheng Road, Taiyuan, Shanxi 030006, China; ^2^College of Chemistry and Chemical Engineering of Shanxi University, No. 92, Wucheng Road, Taiyuan, Shanxi 030006, China

## Abstract

Radix Bupleuri (RB), also named Chaihu in Chinese, is a commonly used herbal drug in traditional Chinese medicine (TCM), and the processing of RB with vinegar to prepare vinegar-baked Radix Bupleuri (VBRB) has a long history in the clinic of TCM. In the present study, GC-MS coupled with multivariate data analysis was applied to compare the volatile components between crude and two vinegar processed RBs. After vinegar baking, the oil yields were decreased significantly, and the chemical compositions were also changed greatly. The chemical changes included the disappearance or appearance, as well as the content increase or decrease of some volatile compounds. The oil yields of two different VBRBs showed no significant difference but differed markedly in their chemical compositions, suggesting that the type of vinegar exerted great impacts on the vinegar-baking process. Thus, the effect of different vinegars on processing should be further investigated to ensure the therapeutic effect and safety of VBRB in clinic.

## 1. Introduction

Radix Bupleuri (“chaihu” in Chinese, short for RB), the dry root of* Bupleurum chinense *DC. or* B. scorzonerifolium *Willd. (Apiaceae), is a commonly used herbal drug in traditional Chinese medicine (TCM) and plays an important role in the treatment of many diseases such as influenza, fever, malaria, hepatitis, jaundice, nephritis, dizziness, lung diseases, cancer, and menstrual disorders in China, Japan, and other Asian countries [1–4]. Chemical investigation of RB revealed the presence of saikosaponins, volatile oils, flavonoids, coumarins, fatty acids, steroids, polysaccharides, and polyacetylenes [5, 6].

Processing of herbal drugs has been a part of the heritage of Chinese medicine for thousands of years; it plays an important role in disease prevention and control for the Chinese people and ensures the safe and effective clinical treatment of TCM [7]. When RB is mixed with vinegar and then baked to dry, it is changed to vinegar-baked Radix Bupleuri (VBRB). The pharmacological effects and components in the drug will change a little bit due to the vinegar-baking procedure. The bile secreting and hepatoprotective effects are enhanced and it is quite effective in curing liver related diseases such as jaundice, hepatitis, cirrhosis, and liver cancer [8–11]. Previous reports showed that the contents of saikosaponin b1 and saikosaponin b2 were increased, while the saikosaponin a, saikosaponin c, and saikosaponin d were decreased [12, 13] after the vinegar-baking process. Previous investigations have demonstrated that the volatile oil of RB showed the effects of anti-influenza, antipyretic, anti-inflammation, and analgesia [14, 15]. After being processed with vinegar, both the yields [16, 17] and compositions [18] of volatile oil in Radix Bupleuri could be changed. In addition, according to Chinese Pharmacopoeia, RB should be baked with rice vinegar to give VBRB. However, there are many kinds of vinegars present in the Chinese market, and most of them were used in the vinegar-baking process of herbal drugs. The influence of different vinegars on the volatile compounds in VBRB remains unknown.

Steam distillation and solvent extraction methods combined with gas chromatography (GC) or gas chromatography-mass spectrometry (GC/MS) are used as the routine methods for the analysis of the volatile oils of TCMs. And GC-MS is one of the most robust methodologies widely applied in volatile metabolite analysis because of its high sensitivity, peak resolution, and reproducibility [19, 20]. Recently, GC-MS-based global metabolic profiling, coupled with multivariate analysis, has been successfully applied to quality assessment of volatile compounds in herbal drugs, such as agarwood [21], Cassia [22], Ginseng [23], and Atractylodis Macrocephalae Rhizoma [24].

In this study, two different vinegars were used to prepare VBRB. And the aim of the present study was to investigate and compare the chemical differences of the volatile oils between crude and vinegar-processed RB samples by GC-MS coupled with multivariate data analysis. Two different VBRBs were also compared to elucidate the vinegar type on vinegar-baking process of RB.

## 2. Materials and Methods

### 2.1. Plant Materials

The Radix Bupleuri was purchased from Shanxi Weikangtang Chinese herbal pieces company and authenticated by Professor Xue-Mei Qin as* B. chinense* DC. A voucher specimen (lot number CH-46) was deposited at Modern Research Center for Traditional Chinese Medicine of Shanxi University.

### 2.2. Solvents and Chemicals

Analytical grade n-hexane was purchased from Beijing Chemical works (Beijing, China) and n-tetracosane (purity > 98%) which used as an internal quality standard for GC-MS analysis was bought from Johnson Matthey Company (Shanghai, China). Bran vinegar was bought from Tongwanzhenji Food Company (Hebei, China) and Shanxi vinegar was from Shanxi Donghu Vinegar Group (Shanxi, China).

### 2.3. VBRB Preparation

According to Chinese Pharmacopoeia, the crude RB (100 g) was incubated with Shanxi vinegar or rice vinegar (20 g), respectively. Then the material was dried by stir-firing to obtain S-VBRB (by Shanxi vinegar) or R-VBRB (by rice vinegar) after vinegar was totally absorbed into raw RB. Six different batches of S-VBRB and R-VBRB were prepared for each kind of vinegars.

### 2.4. Extract of Volatile Oil and GC-MS Analysis

#### 2.4.1. Extraction of Volatile Oil

Steam distillation, a typical extraction method for volatile oils, was chosen according to the Chinese Pharmacopoeia [1]. The dried powder (30 g) was accurately weighed and transferred to a 500 mL round-bottomed flask soaked in 240 mL of water for 2 h. Water was added from the top of the volatile oil determination apparatus until the water spilled onto the round-bottomed flask and 2 mL of n-hexane was added to the water layer. Then the essential oils were extracted by water distillation for 6 h. Volatile oil was separated from the water layer and leached into the n-hexane layer, and then the n-hexane layer was dried over anhydrous sodium sulfate (Na_2_SO_4_) and weighted. The samples were stored at 4°C in the refrigerator before GC-MS analysis. All samples were prepared in sextuplicate.

#### 2.4.2. GC-MS Analysis Parameters

GC-MS analysis was performed using a Polaris Q ion trap mass spectrometer (Thermo Fisher Scientific Inc., USA). Chromatography was performed on a DB-5MS capillary column (30 m × 250 *μ*m i.d., 0.25 *μ*m film thickness; 5% diphenyl cross-linked 95% dimethylpolysiloxane; Agilent J&W Scientific, Folsom, CA). Helium carrier gas was used at a constant flow rate of 1 mL·min^−1^. Approximately 1.0 *μ*L of samples was injected at a constant temperature of 250°C in splitless mode. Initial temperature was set to 50°C and held for 1 min, followed by a ramp to 100°C at 10°C·min^−1^ and held for 2 min and then to 180°C at 3°C·min^−1^, and then rose to 220°C at 15°C·min^−1^ and maintained 1 min and post-run temperature to 300°C for 5 min. The solvent delay was set as 5 min. The interface and source temperatures were set at 280°C and 200°C, respectively. MS detection was implemented with electron ionization (electron energy of 70 eV) and full scan mode (*m/z* 50–650).

#### 2.4.3. Compound Identification

The components eluting within the total ion chromatogram were extracted in AMDIS, matrix interference was then resolved, and overlapping components were removed. Then the compounds were positively identified using the National Institute of Standards and Technology (NIST) 05L Mass Spectra Database containing about 107,000 compounds, as well as comparison with the literatures [18, 25, 26]. The semiquantitative analysis of volatile compounds was performed by comparing their peak areas to that of the internal standard compound on the GC-MS total ion chromatogram. The percentage compositions of compounds were calculated by area normalization method.

#### 2.4.4. Data Analysis

To assess difference (or similarity) between RB and VBRBs, principal component analysis (PCA) was applied to relative peak area values of volatiles obtained on the GC-MS total ion chromatograms using SIMCA-P13.0 (Umetrics, Umeå, Sweden) to clarify the relationship between the RB and VBRBs. In addition, hierarchical heat map clustering analysis was performed with MetaboAnalyst (http://www.metaboanalyst.ca/). The significance level was set at *p* < 0.05 for all tests by SPSS 16.0.

## 3. Results and Discussion

### 3.1. Determination of Volatile Oil Yields

The oil yields were 0.72 ± 0.05, 0.58 ± 0.09, and 0.53 ± 0.05 (mg/g) for RB, S-VBRB, and R-VBRB, respectively. And there was significant difference between the oil yields of crude and processed RBs (*p* < 0.05), while the S-VBRB and R-VBRB showed no significant difference (*p* = 0.18).

### 3.2. Volatile Compounds Identification

**Figure 1:**
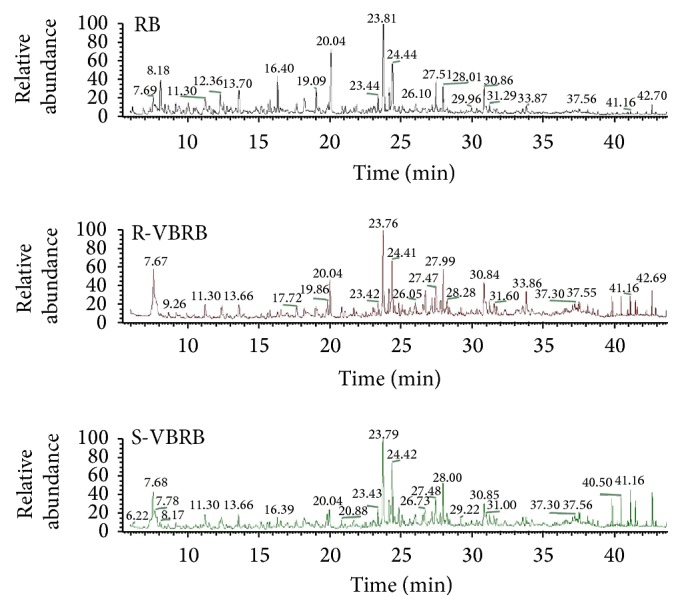
GC-MS chromatograms of raw and processed Radix Bupleuri.

All samples were analyzed by GC-MS, and the TIC chromatograms are shown in Figure 1. A total of 59 compounds were identified in crude and processed RB samples, which amounted for about 75% of the total essential oil, including 15 monoterpenes, 8 sesquiterpenes, 10 aldehydes, 7 phenols (including their esters and ethers), 4 alkane, 3 alcohols, 6 fatty acids, and 6 miscellaneous compounds (Table 1). In the total essential oil, *β*-pinene, 1-methyl-2-isopropyl benzene, 1-methyl-4-(1-methylethyl)-cyclohexadiene, 1-methyl-4-isopropyl benzene, 1-methyl-4-(1-methyethylidene)-cyclohexene, verbenol, 2-(1,1-dimethylethyl)-phenol, 1-isopropyl-2-methoxy-4-toluene, 4-dimethyl-3-cyclohexene-1-acetaldehyde, eucalyptol, menthol, methychavicol, thymol, 2-methyl-5-(1-methylethyl)-phenol, and moslene as the monoterpenes and pentanal, n-hexanal, dodecanal, benzaldehyde, n-nonaldehyde, (E)-2-octenal, (E)-2,4-nonadienal, (E,E)-2,4-sebacic olefin aldehyde, (Z)-2-decenal, and capraldehyde as the aldehydes were determined as the main components.

In addition, 6 compounds (*β*-pinene, 1-methyl-4-(1-methylethyl)-cyclohexadiene, 1,2-cyclooctene oxide, n-nonaldehyde, verbenol, and 2-decenal) found in crude RB samples were disappeared in processed RB samples, while 5 compounds (2,4-dimethoxytoluene, 2-nonyl acetylene, *α*-cubebene, 6-methyl-2-(4-methylphenyl)-5-heptylene, and nerolidol) were newly generated and identified in VBRBs.

### 3.3. Chemical Difference of Raw and Vinegar-Baked RBs by Multivariate Analysis

**Figure 2:**
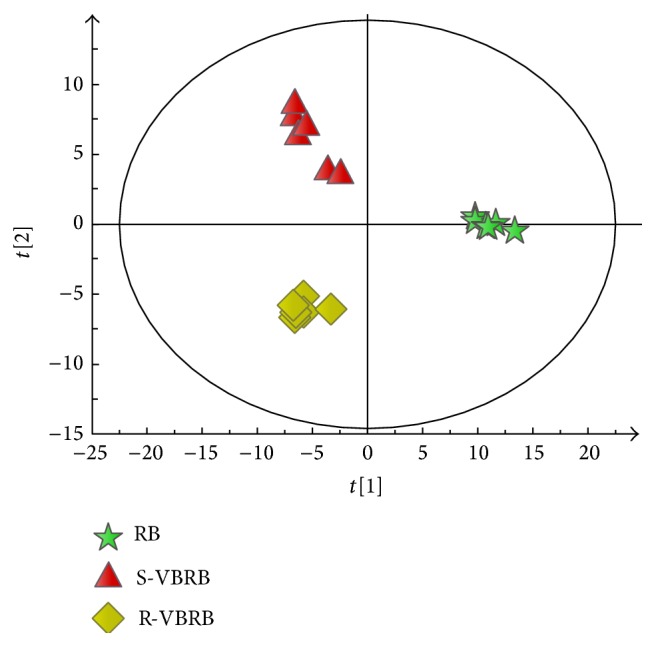
PCA score plots based on GC-MS data of raw Radix Bupleuri (RB) (pentastar), Shanxi vinegar-baked Radix Bupleuri (S-VBRB) (triangle), and rice vinegar-baked Radix Bupleuri (R-VBRB) (diamond).

Since the oils yields of RB decreased significantly after the vinegar-baking process, the change of chemical compositions between raw and processed RBs should be further investigated. Thus, all the GC-MS data (134 peaks) were subjected to PCA analysis to visualize the chemical difference between the raw and vinegar-baked RBs. In the score plot of the first two principal components (PC1: 49.7%, PC2: 20.4%), 18 samples were obviously clustered into three groups (Figure 2). The raw RB was located in the positive side of PC1, while the two vinegar-baked RBs were located on the negative side of PC1, which can be further separated by PC2. The separation between the RB and VBRB was more remarkable than those between the two VBRBs, and the observed separation indicated that RB and VBRB were obviously different in their volatile components.

**Figure 3:**
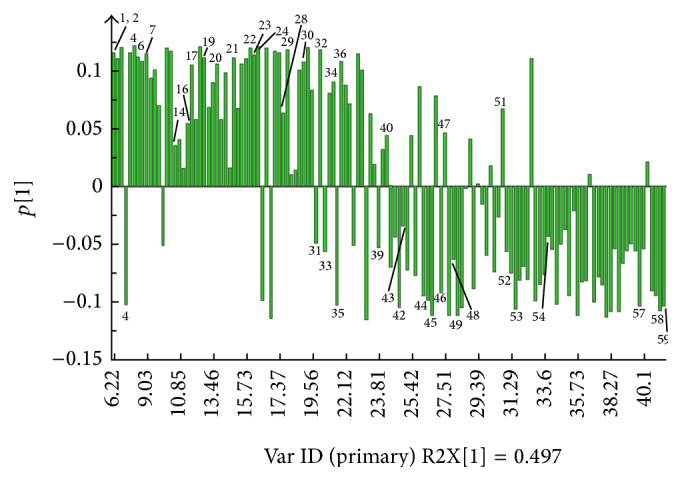
Loading (PC1) plot of PCA results obtained from GC-MS spectra.

The corresponding loadings plot of PC1 (Figure 3) were used to find the components that are responsible for the separation between RB and VBRB. The signals giving a positive effect in PC1 demonstrated that the corresponding metabolites were higher in RB than those in VBRBs. In contrast, the signals with negative values indicated that the level of related components was higher in VBRBs. The signals of pentanal, n-hexanal, 2-amyl furan, dodecanal, 1-methyl-2-isopropyl benzene, 1-methyl-4-isopropyl benzene, 1-methyl-4-(1-methylethylidene)-cyclohexene, (E)-2-octenal, 7-methyl-1-nonyl acetylene, (E)-9-tetradecen-1-ol, 4-ethyl-benzenemethanol, (E,E)-2,4-sebacic olefin aldehyde, guaiacol, 2-(1,1-dimethylethyl)-phenol, 1-(1-cyclohexen-1-yl)-ethanone, 1-isopropyl-2-methoxy-4-toluene, (Z)-2-decenal, p-ethyl guaiacol, capraldehyde, 4-dimethyl-3-cyclohexene-1-acetaldehyde, menthol, 2-methoxy-4-propyl-phenol, (6E)-6-tridecen-4-yne, 1,2-dihydro-acenaphthene, and palmitic acid gave a positive contribution to PC1. The signals with negative values in PC1 included furfural, Z-9-hexadecen-1-ol, eucalyptol, 6-heptyltetrahydro-2H-pyran-2-one, thymol, 6-tert-butyl-2,4-dimethylphenol, 1,3-bis(1-methylethyl)-benzene, 2-methyl-5-(1-methylethyl)-phenol, 1,2-dimethoxy-4-(1-propenyl)-benzene, *α*-ylangene, *α*-guaiene, *β*-ylangene, isoledene, caryophyllene oxide, 9-octadecenoate methyl, moslene, methyl palmitate, stearic acid, and linoleic acid.

**Figure 4:**
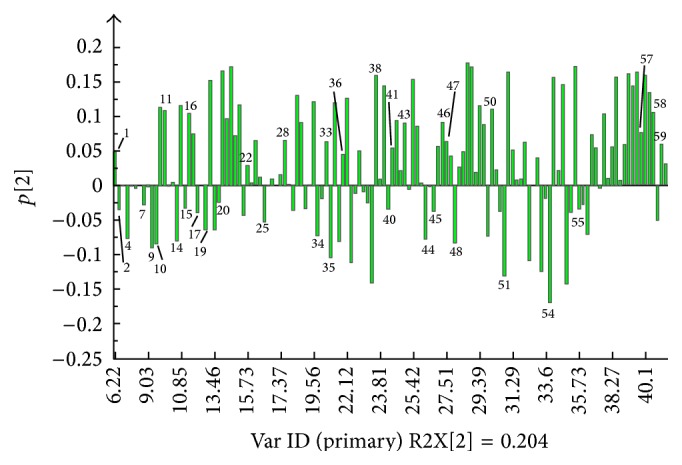
Loading (PC2) plot of PCA results obtained from GC-MS spectra.

The two VBRBs could be clearly separated by PC2. The S-VBRB was grouped on the positive side of PC2, while the R-VBRB was on the negative side of PC2. The corresponding loadings plot (Figure 4) clearly showed that high levels of pentanal, benzaldehyde, 7-methyl-1-nonyl acetylene, guaiacol, 2-(1,1-dimethylethyl)-phenol, (Z)-2-decenal, eucalyptol, 2-methoxy-4-propyl-phenol, methychavicol, 6-tert-butyl-2,4-dimethylphenol, 2-methyl-5-(1-methylethyl)-phenol, *α*-guaiene, 1,2-dihydro-acenaphthene, 1,6,7-trimethylnaphthalene, methyl palmitate, stearic acid, and linoleic acid were present in the S-VBRB. In contrast, higher amounts of n-hexanal, furfural, 1-methyl-2-isopropyl benzene, 1-methyl-4-isopropyl benzene, 1-methyl-4-(1-methylethylidene)-cyclohexene, (E)-2-octenal, (E)-2,4-nonyl diene ether, (E)-9-tetradecen-1-ol, 4-ethyl-benzenemethanol, (E,E)-2,4-sebacic olefin aldehyde, 2,4-dimethoxytoluene, (Z)-9-hexadecen-1-ol, menthol, 6-heptyltetrahydro-2H-pyran-2-one, (6E)-6-tridecen-4-yne, 1,2-dimethoxy-4-(1-propenyl)-benzene, *α*-ylangene, *β*-ylangene, palmitic acid, moslene, and 6-methyl-2-(4-methylphenyl)-5-heptylene were present in R-VBRB.

Table 1 showed the relative contents of identified volatile components in raw and processed RBs, and all the results were shown as mean ± SD. Statistical analysis was carried out using one-way ANOVA by SPSS. The results obtained by quantitative statistical analysis were in agreement with those of multivariate analysis.

**Figure 5:**
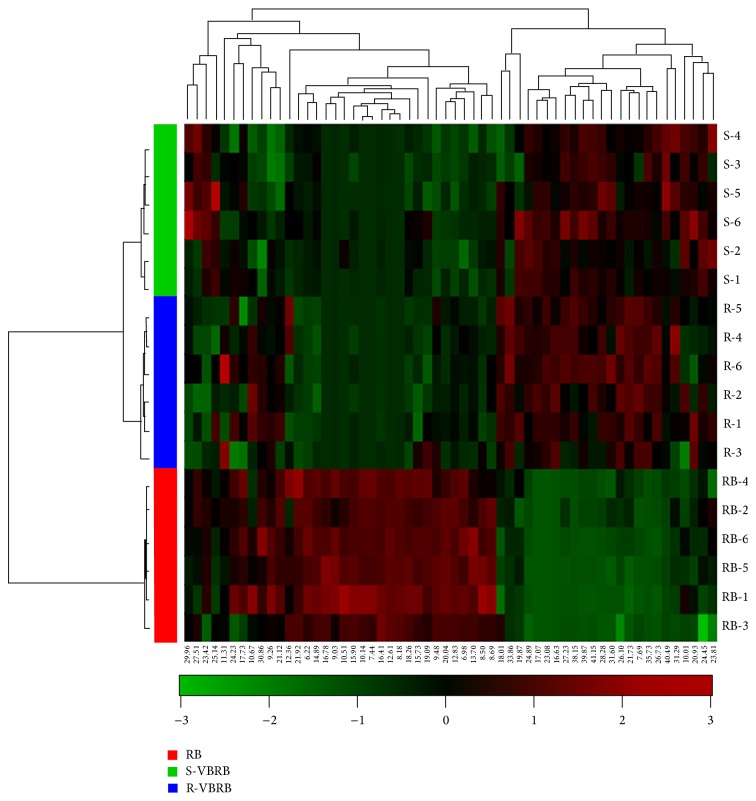
Heat maps of differential components between raw and processed Radix Bupleuri.

To visualize the changes between the raw and two processed RBs, heat map was further generated based on the differential compounds determined above (Figure 5). Here, the red and green colors corresponded to increased and decreased constituents in the VBRB after processing, respectively. It was obvious from the left side that the 18 samples of RB and VBRB could be clearly divided into two main clusters, and S-VBRB and R-VBVB could be separated in the second cluster. The compounds could be also divided into two main clusters on the top, and the left cluster that presented these ingredients were rich in raw RB, while the compounds in the right cluster showed high contents in VBRB.

## 4. Conclusion

In the present study, GC-MS coupled with multivariate data analysis was applied to compare the volatile components between crude and two vinegar-processed RBs. Compared with the previous studies, more chemical components were identified, and the influence of vinegar type on processing was also discussed.

After vinegar-baking, not only the oil yields were decreased significantly, but the chemical compositions were also changed, including the disappearance or appearance, as well as the content increase or decrease of some volatile compounds. According to the theory of TCM, the purpose of herb processing is to increase potency, reduce toxicity and side effects, and alter the properties or functions [27]. The relationship between the change of volatile oil in RB and the drug action should be further investigated.

Two different VBRBs also showed differences in the volatile compositions, suggesting that the type of vinegar exerted great impacts on the vinegar-baking process. Thus, in order to ensure the therapeutic effect and safety of VBRB in clinic, the effect of processing by different vinegars should be further investigated on the other herbal drugs.

## Figures and Tables

**Table 1:** Volatile compounds and the relative contents in raw and processed Radix Bupleuri (*n* = 6).

No.	*t*_*R*_/min	Compound	Formula	Relative content/%		
				RB	S-VBRB	R-VBRB
1	6.22	Pentanal	C_5_H_10_O	0.41 ± 0.06	0.11 ± 0.02^*∗∗∗*^	—
2	6.98	n-Hexanal	C_6_H_12_O	0.55 ± 0.05	0.18 ± 0.06^*∗∗∗*^	0.26 ± 0.04^*∗∗∗*,#^
3	7.44	*β*-Pinene	C_10_H_16_	0.34 ± 0.07	—	—
4	7.69	Furfural	C_5_H_4_O_2_	2.83 ± 0.38	6.46 ± 1.36^*∗∗∗*^	9.12 ± 1.06^*∗∗∗*,##^
5	8.18	2-Amyl furan	C_9_H_14_O	3.31 ± 0.17	0.33 ± 0.02^*∗∗∗*^	0.40 ± 0.02^*∗∗∗*,###^
6	8.50	Dodecanal	C_12_H_24_O	0.46 ± 0.12	0.18 ± 0.01^*∗∗*^	0.18 ± 0.06^*∗∗*^
7	8.69	1-Methyl-2-isopropyl benzene	C_10_H_14_	0.81 ± 0.12	0.46 ± 0.06^*∗∗∗*^	0.52 ± 0.06^*∗∗∗*^
8	9.03	1-Methyl-4-(1-methylethyl)-cyclohexadiene	C_10_H_16_	0.21 ± 0.02	—	—
9	9.26	1-Methyl-4-isopropyl benzene	C_10_H_14_	0.71 ± 0.13	0.36 ± 0.05^*∗∗*^	0.53 ± 0.05^*∗*,#^
10	9.48	1-Methyl-4-(1-methylethylidene)-cyclohexene	C_10_H_16_	0.89 ± 0.08	0.34 ± 0.03^*∗∗∗*^	0.59 ± 0.11^*∗∗∗*,##^
11	10.01	Benzaldehyde	C_7_H_6_O	0.42 ± 0.04	0.55 ± 0.03^*∗∗∗*^	0.44 ± 0.08^#^
12	10.14	1,2-Cyclooctene oxide	C_8_H_14_O	0.80 ± 0.16	—	—
13	10.51	n-Nonaldehyde	C_9_H_18_O	0.65 ± 0.09	—	—
14	10.67	(E)-2-Octenal	C_8_H_14_O	0.55 ± 0.12	0.43 ± 0.07	0.54 ± 0.13
15	11.31	(E)-2,4-Nonadienal	C_9_H_14_O	1.73 ± 0.07	1.58 ± 0.11^*∗*^	1.85 ± 0.22
16	11.64	7-Methyl-1-nonyl acetylene	C_10_H_18_	0.69 ± 0.04	0.68 ± 0.07	0.43 ± 0.02^*∗*,#^
17	12.36	(E)-9-Tetradecen-1-ol	C_14_H_28_O	2.69 ± 0.40	2.15 ± 0.20^*∗*^	2.37 ± 0.60
18	12.61	Verbenol	C_10_H_16_O	0.79 ± 0.04	—	—
19	12.83	4-Ethyl-benzenemethanol	C_9_H_12_O	0.83 ± 0.03	0.30 ± 0.05^*∗∗∗*^	0.51 ± 0.03^*∗∗∗*,###^
20	13.70	(E,E)-2,4-Sebacic olefin aldehyde	C_10_H_16_O	2.09 ± 0.15	1.22 ± 0.16^*∗∗∗*^	1.34 ± 0.21^*∗∗∗*^
21	14.89	Guaiacol	C_7_H_8_O_2_	1.31 ± 0.09	0.74 ± 0.07^*∗∗∗*^	0.37 ± 0.05^*∗∗∗*,###^
22	15.73	2-(1,1-Dimethylethyl)-phenol	C_10_H_14_O	0.79 ± 0.05	0.56 ± 0.05^*∗∗∗*^	0.50 ± 0.10^*∗∗∗*^
23	15.90	1-(1-Cyclohexen-1-yl)-ethanone	C_8_H_12_O	1.00 ± 0.09	0.52 ± 0.06^*∗∗∗*^	0.51 ± 0.04^*∗∗∗*^
24	16.41	1-Isopropyl-2-methoxy-4-toluene	C_11_H_16_O	3.19 ± 0.31	0.74 ± 0.06^*∗∗∗*^	0.52 ± 0.11^*∗∗∗*,##^
25	16.63	2,4-Dimethoxytoluene	C_9_H_12_O_2_	—	0.67 ± 0.13	0.91 ± 0.23
26	16.78	2-Decenal	C_10_H_12_O_2_	0.4 ± 0.08	—	—
27	17.07	2-Nonyl acetylene	C_9_H_16_	—	0.68 ± 0.09	0.62 ± 0.10
28	17.73	(Z)-2-Decenal	C_10_H_18_O	1.07 ± 0.15	0.96 ± 0.05	0.82 ± 0.19^*∗*^
29	18.26	p-Ethyl guaiacol	C_9_H_12_O_2_	1.73 ± 0.11	0.95 ± 0.21^*∗∗∗*^	0.82 ± 0.10^*∗∗∗*^
30	19.09	Capraldehyde	C_10_H_20_O	2.24 ± 0.13	1.41 ± 0.29^*∗∗∗*^	1.46 ± 0.21^*∗∗*^
31	19.87	(Z)-9-Hexadecen-1-ol	C_16_H_32_O	1.16 ± 0.09	1.37 ± 0.28	1.39 ± 0.14^*∗*^
32	20.04	4-Dimethyl-3-cyclohexene-1-acetaldehyde	C_10_H_16_O	6.02 ± 0.77	2.01 ± 0.38^*∗∗∗*^	2.46 ± 0.36^*∗∗∗*^
33	20.93	Eucalyptol	C_10_H_18_O	0.60 ± 0.02	0.72 ± 0.06^*∗∗*^	0.66 ± 0.10
34	21.12	Menthol	C_10_H_20_O	0.50 ± 0.04	0.32 ± 0.06^*∗∗∗*^	0.43 ± 0.06^*∗*,#^
35	21.73	6-Heptyltetrahydro-2H-pyran-2-one	C_12_H_22_O_2_	0.48 ± 0.07	0.68 ± 0.06^*∗∗∗*^	0.89 ± 0.08^*∗∗∗*,###^
36	21.92	2-Methoxy-4-propyl-phenol	C_10_H_14_O_2_	0.86 ± 0.14	0.43 ± 0.05^*∗∗∗*^	0.28 ± 0.05^*∗∗∗*,##^
37	23.08	*α*-Cubebene	C_15_H_24_	—	0.97 ± 0.19	1.13 ± 0.10
38	23.42	Methychavicol	C_10_H_12_O	1.31 ± 0.18	1.49 ± 0.08	1.02 ± 0.08^*∗*,###^
39	23.81	Thymol	C_10_H_14_O	8.05 ± 0.44	8.63 ± 0.42^*∗*^	8.37 ± 0.18
40	24.23	(6E)-6-Tridecen-4-yne	C_13_H_22_	1.36 ± 0.16	1.22 ± 0.13	1.27 ± 0.17
41	24.45	6-Tert-butyl-2,4-dimethylphenol	C_12_H_18_O	4.12 ± 0.49	4.86 ± 0.23^*∗*^	4.54 ± 0.21^#^
42	24.89	1,3-Bis(1-methylethyl)-benzene	C_12_H_18_	0.53 ± 0.07	1.36 ± 0.26^*∗∗∗*^	1.22 ± 0.12^*∗∗∗*^
43	25.14	2-Methyl-5-(1-methylethyl)-phenol	C_10_H_14_O	1.07 ± 0.05	1.22 ± 0.15	1.07 ± 0.11
44	26.10	1,2-Dimethoxy-4-(1-propenyl)-benzene	C_11_H_14_O_2_	1.14 ± 0.35	1.81 ± 0.17^*∗∗*^	2.39 ± 0.27^*∗∗∗*,##^
45	26.73	*α*-Ylangene	C_15_H_24_	0.46 ± 0.04	1.35 ± 0.22^*∗∗∗*^	1.66 ± 0.30^*∗∗∗*^
46	27.23	*α*-Guaiene	C_15_H_24_	0.89 ± 0.10	1.83 ± 0.39^*∗∗*^	1.76 ± 0.36^*∗∗*^
47	27.51	1,2-Dihydro-acenaphthene	C_12_H_10_	3.05 ± 0.42	3.29 ± 0.59	2.58 ± 0.30^*∗*,#^
48	28.01	*β*-Ylangene	C_15_H_24_	2.32 ± 0.26	2.58 ± 0.44	2.95 ± 0.18^*∗∗*^
49	28.28	Isoledene	C_15_H_24_	0.68 ± 0.06	1.60 ± 0.26^*∗∗∗*^	1.49 ± 0.26^*∗∗∗*^
50	29.96	1,6,7-Trimethylnaphthalene	C_13_H_14_	0.68 ± 0.04	0.92 ± 0.33	0.56 ± 0.14^#^
51	30.86	Palmitic acid	C_16_H_32_O_2_	3.85 ± 0.57	2.27 ± 0.47^*∗∗∗*^	3.17 ± 0.39^#^
52	31.29	Caryophyllene oxide	C_15_H_24_O	0.88 ± 0.12	1.32 ± 0.37^*∗*^	1.36 ± 0.23^*∗*^
53	31.60	9-Octadecenoate methyl	C_19_H_36_O_2_	0.49 ± 0.06	0.90 ± 0.13^*∗∗∗*^	0.90 ± 0.15^*∗∗∗*^
54	33.86	Moslene	C_10_H_16_	0.97 ± 0.12	0.98 ± 0.05	2.96 ± 0.51^*∗∗∗*,###^
55	35.73	6-Methyl-2-(4-methylphenyl)-5-heptylene	C_15_H_22_	—	0.18 ± 0.03	0.20 ± 0.02
56	38.15	Nerolidol	C_15_H_26_O	—	0.45 ± 0.09	0.44 ± 0.10
57	39.87	Methyl palmitate	C_17_H_34_O_2_	0.19 ± 0.05	1.14 ± 0.31^*∗∗∗*^	0.96 ± 0.23^*∗∗∗*^
58	40.49	Stearic acid	C_18_H_36_O_2_	0.05 ± 0.03	1.02 ± 0.43^*∗∗∗*^	0.49 ± 0.08^*∗∗∗*,#^
59	41.15	Linoleic acid	C_18_H_32_O_2_	0.13 ± 0.05	1.07 ± 0.20^*∗∗∗*^	0.83 ± 0.13^*∗∗*^

*∗* means compared to RB (^*∗*^ *p* < 0.05, ^*∗∗*^ *p* < 0.01, ^*∗∗∗*^ *p* < 0.001); # means compared to S-VBRB (^#^ *p* < 0.05, ^##^ *p* < 0.01, ^###^ *p* < 0.001).
